# Improving nerve and muscle function: an exploration of targeted nerve function replacement following differential delay periods in a rat model

**DOI:** 10.1186/s12984-025-01666-0

**Published:** 2025-07-04

**Authors:** Chunxiao Tang, Yuanheng Li, Xinxian Fan, Jiamei Guo, Yifeng Lin, Yifan Gao, Lin Yang

**Affiliations:** 1 Zhuhai Campus of Zunyi Medical University, Zhuhai, Guangdong Province 519090 China; 2 Neural Engineering Center, Shenzhen Institutes of Advanced Technology, Chinese Academy of Sciences, Shenzhen, Guangdong Province 518000 China; 3 School of Intelligent Medical Engineering, Shenzhen Technology University, Shenzhen, Guangdong Province 518000 China; 4 Department of Human Anatomy and Embryology, School of Basic Medicine Science, Putian University, Putian, Fujian Province 351100 China

**Keywords:** Targeted nerve function replacement (TNFR), Electromyography (EMG), Autophagic behavior, Spinal cord, Dorsal Root Ganglion (DRG)

## Abstract

**Background:**

Targeted Muscle Reinnervation (TMR) improves real-time control of EMG-based prostheses by connecting severed nerves to adjacent muscles, creating new EMG signals. However, TMR requires cutting original nerve connections, which can cause denervation atrophy and limit functional recovery. As an alternative, Targeted Nerve Function Replacement (TNFR) offers a fundamentally different approach by establishing a direct end-to-end anastomosis between an intact donor nerve and the original nerve of a target muscle, preserving existing neural pathways while providing supplementary neural input. This study evaluates TNFR efficacy in restoring denervated muscle function across different postoperative intervals in a rat model.

**Methods:**

Thirty Sprague–Dawley rats (220–250 g) were divided into five equal groups (n = 6 per group): control (no transection), denervated (transection without repair), immediate TNFR after median nerve transection, 2-week delayed TNFR, and 4-week delayed TNFR. The median nerve was selected for reinnervation with the musculocutaneous nerve innervating the brachialis muscle serving as the anastomosis target. All assessments were conducted 4 weeks post-TNFR intervention, including intramuscular bipolar EMG recordings (1024 Hz sampling rate), behavioral assessment, muscle tension measurement, dorsal root ganglia (DRG) histology, and spinal cord motor neuron evaluation.

**Results:**

Immediate TNFR significantly outperformed delayed interventions across all parameters. EMG amplitude and root mean square values were significantly higher in the immediate group (P < 0.05). Maximum contraction and tetanic contraction forces of biceps brachii showed superior recovery with immediate TNFR (P < 0.05). Histological examination revealed greater preservation of DRG sensory neurons following TNFR (P < 0.05). Immunofluorescence showed better preservation of synaptic protein expression in spinal cord motor neurons with immediate intervention. Immediate TNFR also prevented autophagic behavior seen in delayed intervention groups, suggesting improved neuropathic pain prevention.

**Conclusion:**

Timing critically influences TNFR outcomes, with immediate intervention yielding optimal restoration of both motor and sensory functions. This study provides valuable insights for optimizing surgical strategies in peripheral nerve injury, with important implications for limb reconstruction, rehabilitation protocols, and prosthetic development.

**Graphical abstract:**

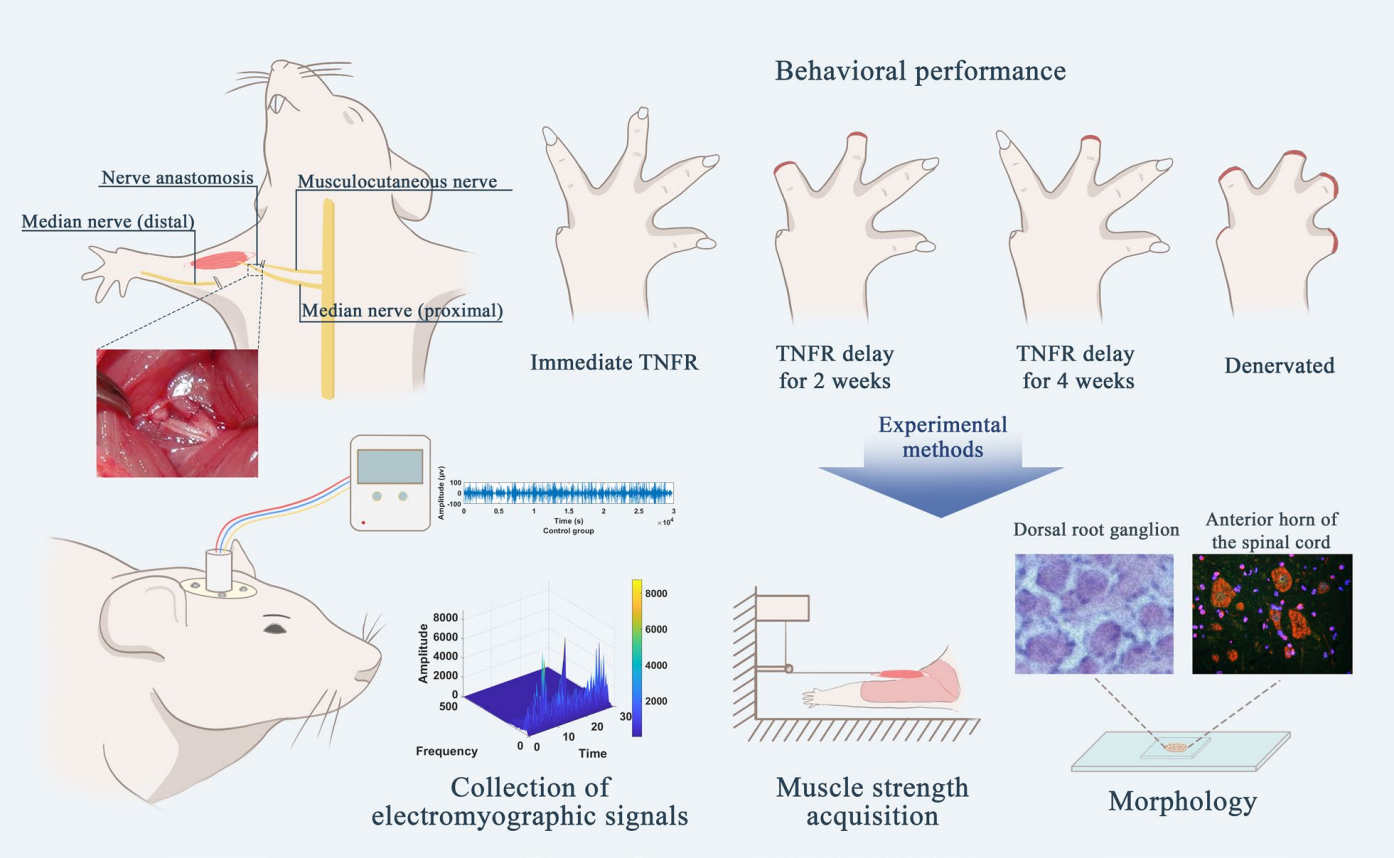

**Supplementary Information:**

The online version contains supplementary material available at 10.1186/s12984-025-01666-0.

## Introduction

Maintaining limb functionality is essential for seamless interaction with the environment. However, traumatic injuries, vascular diseases, and neurological disorders can severely impair mobility, leading to chronic pain and psychological distress [1]. Current commercial prosthetics often suffer from limited functionality and delayed response due to inadequate electromyography (EMG) signal sources, resulting in awkward and inefficient movements. This highlights the urgent need for multifunctional prosthetics with intuitive control, particularly for high-level amputees, which can be achieved through advanced surgical interventions.

Kuiken et al. introduced a innovative surgical technique known as Targeted Muscle Reinnervation (TMR) [2–4]. This approach involves redirecting nerves from the residual limb to specific muscles, necessitating the disconnection of their original neural connections. While TMR effectively restores motor signals and facilitates EMG collection from target muscles, it introduces significant drawbacks including denervation atrophy of original muscle targets, potential neuroma formation at transection sites, and limited sensory recovery. These limitations can compromise long-term functional outcomes and rehabilitation potential. In TMR procedures, this surgical approach enables regenerated nerve axons to produce action ability — electrical signals that activate muscles in the residual limb, thereby restoring function [5–7]. Subsequently, limb muscles serve as a pathway for nerve signals to reach the skin surface, generating new EMG signals that can enhance prosthetic control [8]. TMR technology holds promise for improving EMG prosthetic functionality by offering the capability to rehabilitate joint movements and enabling intricate multi-joint motions [9, 10]. Moreover, TMR shows capacity in restoring movement capabilities and alleviating discomfort in lower limb amputees, fostering optimism for fully functional prosthetic limbs in the future [10–12].

Compared to the well-established TMR technique, Targeted Nerve Function Replacement (TNFR) offers a novel approach by directly reconnecting the target nerve to its original neural pathway, which may lead to superior outcomes in preserving motor and sensory functions while minimizing denervation-induced atrophy. However, TMR surgery involves severing the original neural connections of the target muscle, which can disrupt the normal activity of proteins and metabolic enzymes within those muscles. Additionally, the severed nerve endings undergo Wallerian degeneration, gradually breaking down over time due to a lack of stimulation from the neuron cell body. This degeneration impairs the ability of regenerated axons or nerve fibers to effectively communicate with distant denervated skeletal muscles, exacerbating muscle weakness and atrophy. During this phase, skeletal muscle volume decreases, leading to a significant reduction or complete loss of contraction function. Muscle fibers may develop severe fibrosis and risk irreversible atrophy, ultimately resulting in the loss of motor and sensory functions [11, 13]. To address these challenges, researchers have developed several innovative techniques for nerve repair and reinnervation. It is important to distinguish between three key approaches that have emerged in this field: TMR, Regenerative Peripheral Nerve Interfaces (RPNI), and TNFR. TMR, as previously described, redirects severed nerves from the amputated limb to new target muscles after deliberately denervating those muscles. While effective for prosthetic control, this approach sacrifices the original innervation of the target muscles, with the likelihood of leading to complications associated with denervation. RPNI employs a different strategy, utilizing free muscle grafts that are carefully wrapped around the terminal ends of transected peripheral nerves [14, 15]. These grafts function as biological amplifiers for nerve signals, providing stable interfaces between peripheral nerves and artificial limbs. Unlike TMR, which involves redirecting nerves to muscles that may no longer serve their original biomechanical function, RPNI does not require altering the innervation of healthy remaining muscles. TNFR, the central focus of this study, represents a fundamentally distinct approach. Rather than redirecting nerves to new muscles (as in TMR) or using muscle grafts (as in RPNI), TNFR establishes a direct end-to-end anastomosis between an intact donor nerve and the original nerve of a target muscle. This sophisticated technique preserves existing neural pathways while providing supplementary neural input to the target muscle.

**Fig. 1:**
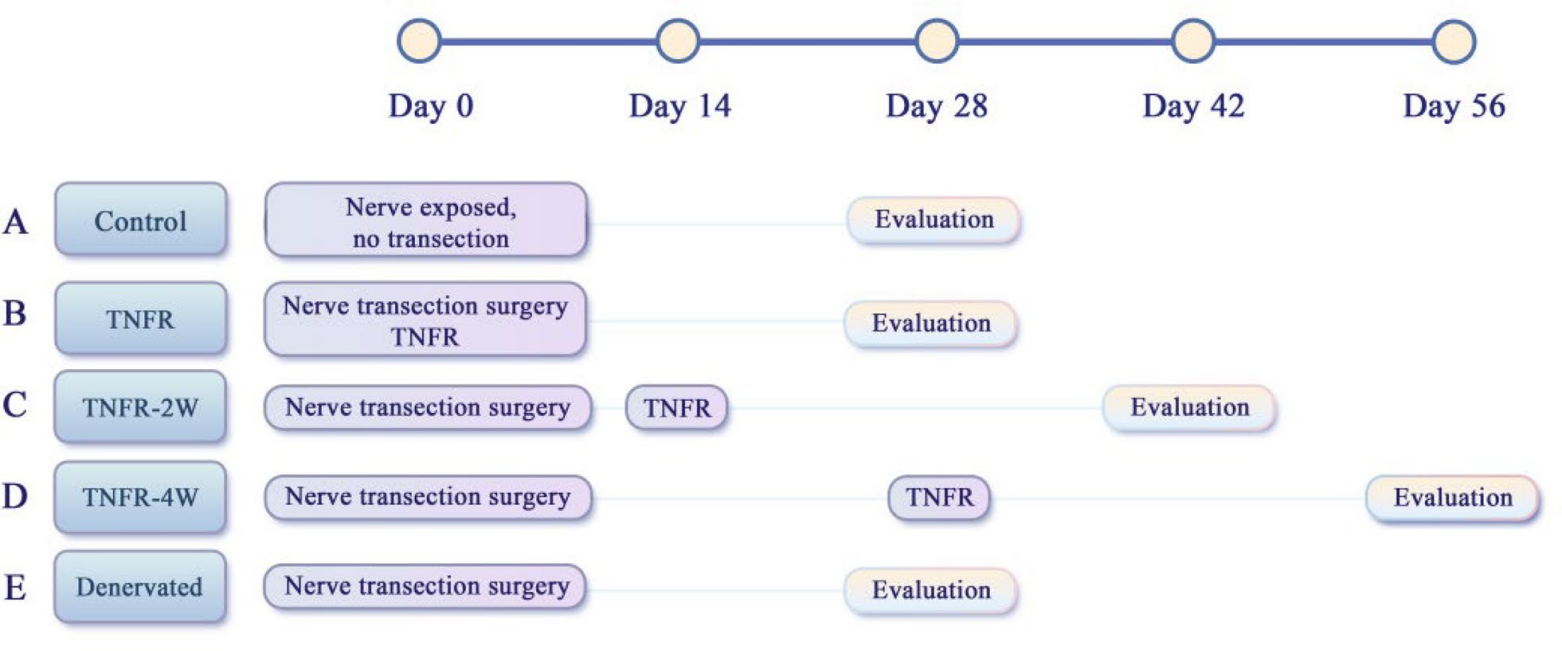
Experimental timeline comparing the effects of TNFR therapy timing on nerve regeneration. **A** The control group underwent nerve exposure but no nerve transection and was evaluated on day 28. **B** The TNFR group received treatment at the same time as the nerve transection surgery and was evaluated on day 28. **C** The 2-week delayed (TNFR-2W) group received intervention on day 14 and was evaluated on day 42. **D** The 4-week delayed (TNFR-4W) group received treatment on day 28 and was evaluated on day 56. **E** The denervated group underwent nerve transection on day 0 and was evaluated on day 28. All groups were evaluated 28 days after intervention to assess how treatment delays affected functional recovery outcomes

In our precisely engineered TNFR model, we perform a meticulous microsurgical procedure where the median nerve serves as the donor nerve and connects to the musculocutaneous nerve that innervates the brachialis muscle. The anastomosis is strategically positioned at the nerve entry point to the target muscle, as illustrated in Fig. 1. This critical positioning protects motor neurons from prolonged denervation by maintaining neural connections to the target muscle while redirecting prospective damage away from the proximal regions containing vital motor neuron bodies toward more distal nerve segments.

The TNFR procedure begins with careful exposure of both the donor nerve (median nerve) and the recipient nerve (musculocutaneous nerve) near its entry point to the brachialis muscle. The recipient nerve undergoes proximal transection, while the donor nerve is partially transected to create a specialized branch for anastomosis, thereby preserving a portion of its original function. This strategic partial transection creates an effective neural pathway that enables signals from the donor nerve to reach the denervated muscle, as depicted in Fig. 3. TNFR preserves muscle viability and function after significant injury by maintaining some original nerve function while adding new neural input. The timing of TNFR intervention likely affects recovery outcomes, much like the established "golden period" for nerve repair [16, 17]. Although this concept is widely discussed, there remains ongoing debate regarding its exact timeframe, suggesting that in clinical practice, the timing of nerve repair may need to be customized based on individual circumstances. Despite recent advances, the specific impact of TNFR surgery timing on functional outcomes remains incompletely understood, necessitating further research to determine optimal intervention windows for maximizing recovery potential. TNFR involves creating an anastomosis between the target nerve and the original nerve of the target muscle, rather than simply implanting the target nerve into the muscle. This method facilitates reinnervation of the target muscle by its original nerve, thereby restoring both motor and sensory functions. Additionally, precise and consistent surgical incisions during nerve repair procedures generally promote faster wound healing and better functional outcomes [18, 19]. While studies on related techniques such as RPNI have demonstrated the benefits of a careful surgical approach [15, 20], further investigation is required to establish its long-term efficacy. TNFR’s unique methodology–connecting an intact donor nerve to the recipient nerve near the muscle entry point–warrants specific investigation of its wound healing dynamics. The distinct neural network structure within targeted muscles may enhance reinnervation rates following TNFR. However, various clinical conditions can delay nerve repair interventions, potentially compromising outcomes if the optimal ‘golden period’ for neural regeneration is missed. This strategic positioning near the muscle entry point helps maintain the nerve’s connection to its target muscle, thereby shifting the focus of potential damage to the more distal segments of the nerve rather than the proximal regions containing essential motor neuron structures. This adjustment extends the critical window for effective nerve repair and possibility improves the likelihood of successful reinnervation and functional recovery.

This study aimed to comprehensively evaluate the recovery of sensory and motor functions in rats that underwent TNFR surgery at different time points after injury. We established a standardized TNFR model using the median nerve for reinnervation and the musculocutaneous nerve innervating the brachialis muscle for anastomosis. Following surgery, we conducted a multifaceted assessment of recovery through intramuscular EMG signal analysis, precise muscle tension measurements, detailed behavioral assessments, quantitative sensory neuron counts in dorsal root ganglia (DRG), and comprehensive motor neuron evaluation in the spinal cord.

## Materials and methods

### Animals and experimental design

The selection of Sprague–Dawley rats was based on their extensive use in nerve injury models, which provides a consistent framework for evaluating TNFR interventions. Although this strain is prone to autotomy behaviors post-injury, their high availability and well-characterized physiology make them a preferred model for initial exploratory studies. To address autotomy-related limitations, postoperative care protocols were strictly implemented. Additionally, the experimental design aimed to minimize variability by using age-matched and weight-matched subjects under controlled environmental conditions. Thirty Specific Pathogen-Free (SPF) adult male Sprague-Dawley rats, aged 7-8 weeks with a body weight of 220-250 g, were purchased from the Guangdong Medical Laboratory Animal Center, Guangzhou, China (license No. SCXK (Yue) 2013-0002). Sample size determination was based on power analysis using G*Power software (version 3.1). With an anticipated effect size of 0.6 (based on preliminary data and previous studies), alpha of 0.05, and desired power of 0.8 for one-way ANOVA comparisons across five groups, a minimum sample size of 6 animals per group was required. For EMG signal analyses where multiple measurements were collected per animal, the repeated sampling provided increased statistical power while maintaining the same number of animals. This sample size accounts for potential variability in outcome measures while minimizing animal usage in accordance with ethical principles and ARRIVE guidelines. The rats were kept in an SPF environment at the Zhuhai campus of Zunyi Medical University, Zhuhai, China. The rats were housed in cages (n = 3/cage) under controlled conditions, with a temperature of 22-26℃, relative humidity of 40–60%, and a 12:12 h light–dark cycle, with free access to food and water. The rats were randomly divided into five groups: control, denervated, TNFR, 2-week delayed TNFR (TNFR-2W), and 4-week delayed TNFR (TNFR-4W), with six rats in each group. The study protocol was approved by the Animal Ethics Committee of the Zhuhai campus of Zunyi Medical University, Zhuhai, China (approval No. 2019-2-273) on March 11, 2019. The study was conducted in accordance with the ARRIVE 2.0 guidelines (Animal Research: Reporting of In Vivo Experiments) [21].

#### Experimental groups and timeline

The experiment was designed to evaluate the effectiveness of TNFR at different time points following denervation. Figure 1 illustrates the experimental timeline for all groups. After acclimatization, all experimental groups except the Control underwent median nerve transection on Day 0. The TNFR group received the TNFR procedure immediately after nerve transection. The TNFR-2W group received delayed TNFR surgery 2 weeks after nerve transection (Day 14), while the TNFR-4W group received delayed TNFR surgery 4 weeks after nerve transection (Day 28). Functional and histological evaluations were conducted at 4 weeks post-TNFR surgery for all groups.

### Surgical procedures

**Fig. 2:**
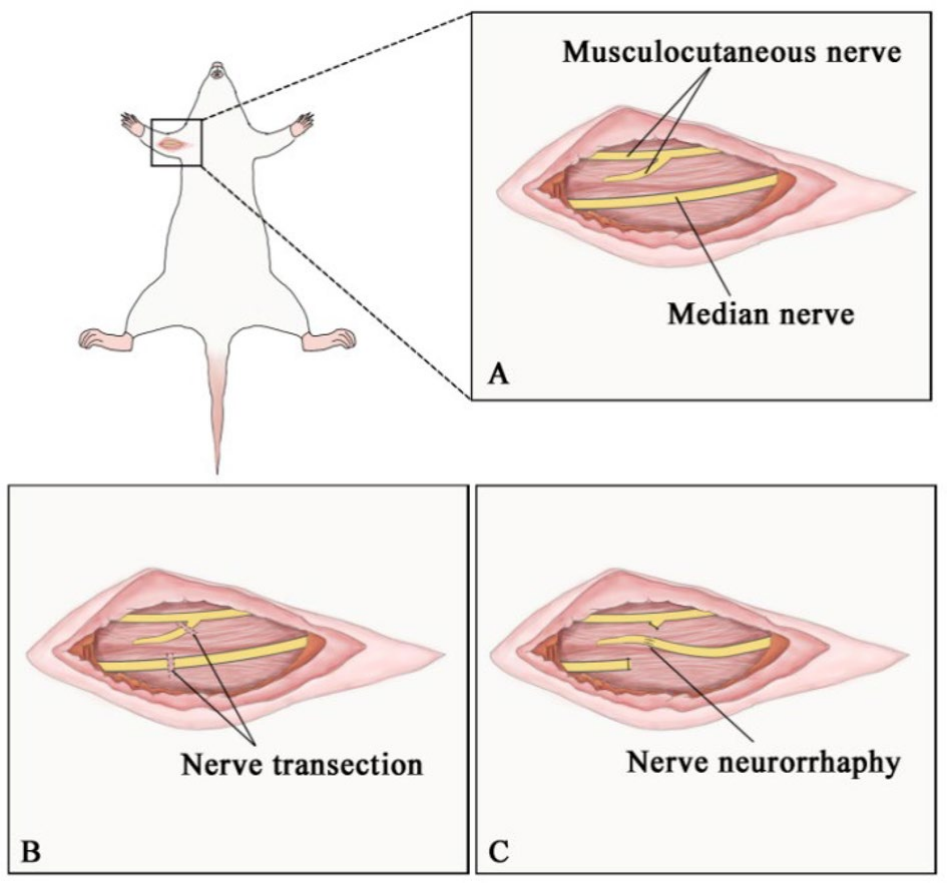
Surgical procedures for the TNFR model in rats. **A** Anatomical diagram showing the location of the median nerve and musculocutaneous nerve in the ventral right forelimb of the rat. **B** Nerve transection surgery showing complete transection of the proximal musculocutaneous nerve and the distal median nerve. **C** Nerve suture surgery showing the end-to-end anastomosis between the proximal musculocutaneous nerve and the distal median nerve to establish the TNFR model

A TNFR model was established following the TMR surgery protocol described by Jianping Huang et al. [22]. Rats were deprived of food for 12 h and water for 4 h prior to surgery. This fasting protocol was implemented to minimize the risk of aspiration during anesthesia, a standard precaution in rodent surgical procedures. Perioperative analgesia was administered to ensure animal welfare. Buprenorphine (0.05 mg/kg) was given subcutaneously 30 min prior to surgery to manage pain and provide prolonged analgesic effects. Additional doses were administered every 12 h postoperatively for 48 h to ensure adequate pain relief during the recovery period. Anesthesia was induced with 3% isoflurane for approximately 3-5 min and maintained at 2% isoflurane throughout the operation. The anesthetized rats were fixed in a supine position on the operating table, and the surgical area was fully exposed and depilated for skin preparation. The right ventral side of each rat was used as the experimental side. For the TNFR procedure, the right median nerve was anastomosed with the musculocutaneous nerve, and recording electrodes were implanted in the biceps brachii. The left ventral side was used as the normal side, receiving only electrode implantation with no nerve manipulation. The area was disinfected with povidone-iodine and then deiodinated with 75% alcohol after drying. A surgical incision was made 1.5-2 cm perpendicular to the midline of the elbow from the acromion. The skin, superficial fascia (subcutaneous tissue), and deep fascia were sequentially opened to isolate the musculocutaneous nerve and the connective tissue adjacent to the median nerve. For the TNFR procedure, once the nerve was fully exposed, the proximal end of the musculocutaneous nerve and the distal end of the median nerve were ligated with an 8-0 suture and severed. An end-to-end anastomosis was performed as close as possible to the point where the biceps nerve enters the muscle, followed by disinfection and layer-by-layer suturing of the surgical area. (Fig. 2). In the control group, the nerves were exposed but no transection or anastomosis was performed. In the denervation group, the nerves were transected without subsequent anastomosis. For the delayed treatment groups, the TNFR-2W group received anastomosis 2 weeks post-denervation, while the TNFR-4W group received anastomosis 4 weeks post-denervation.

#### Implantation of EMG electrodes in rats

We surgically implanted EMG electrodes into the rats’ bilateral biceps brachii muscles. Each rat was positioned prone and secured using a stereotactic device, with an incision made to expose the skull and remove the fascia. A stereotactic device was then implanted and anchored in the skull of each rat to ensure precise and stable placement of electrodes for chronic neural and muscle activity recordings. This setup allows for accurate targeting of specific anatomical sites and minimizes movement-related artifacts, which is essential for obtaining reliable, consistent data across experimental sessions. By securing the device within the skull, we prevented any shifting or dislodgement, which could compromise data quality and animal safety.

**Fig. 3:**
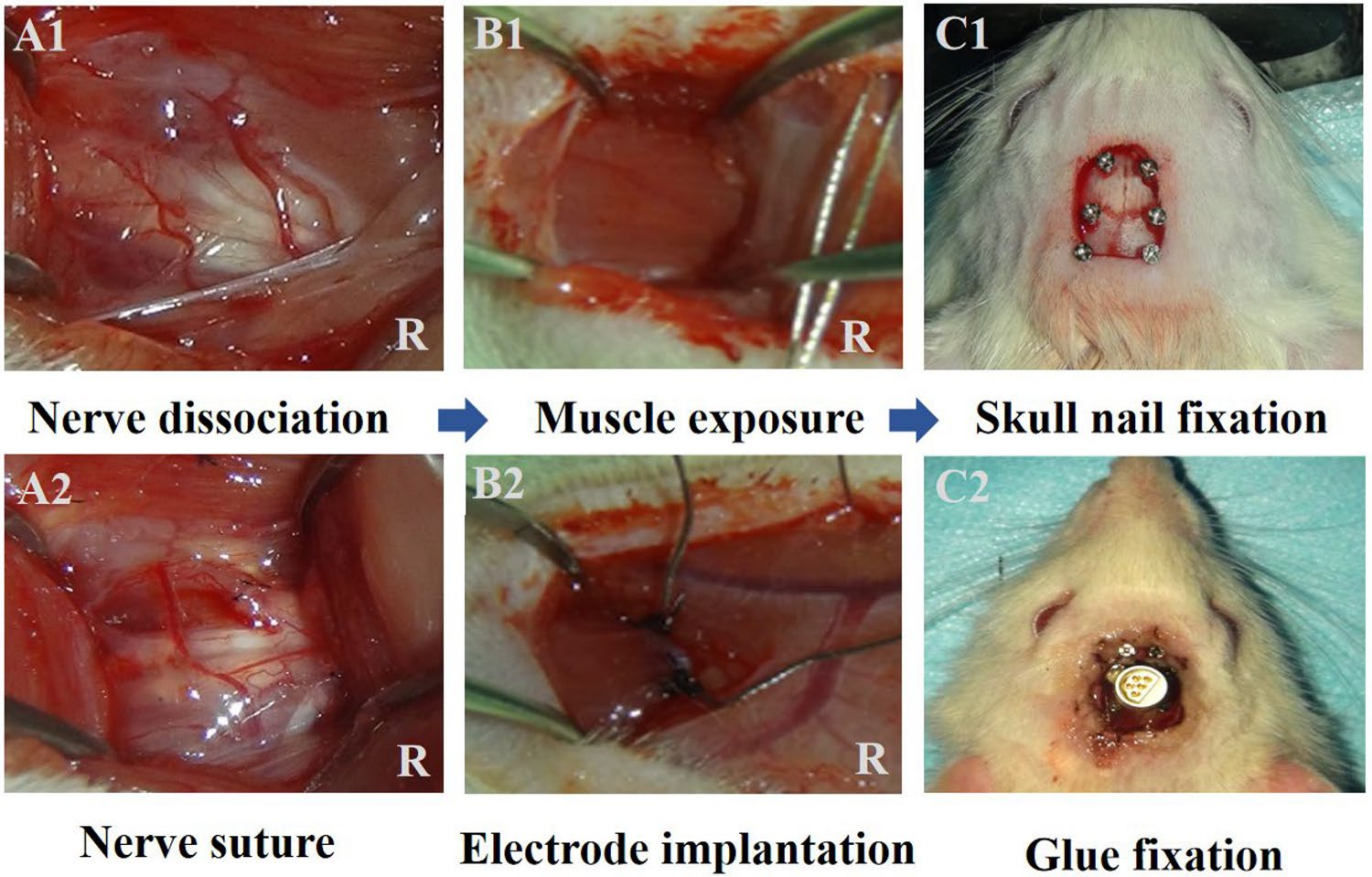
External skull joint fixation. **A1** and **A2** Separation and suture of the nerves. **B1** Biceps exposure and electrode penetration. **B2** Recording electrode implantation. **C1** Skull exposure and skull nail fixation. **C2** Skull joint fixation

**Fig. 4:**
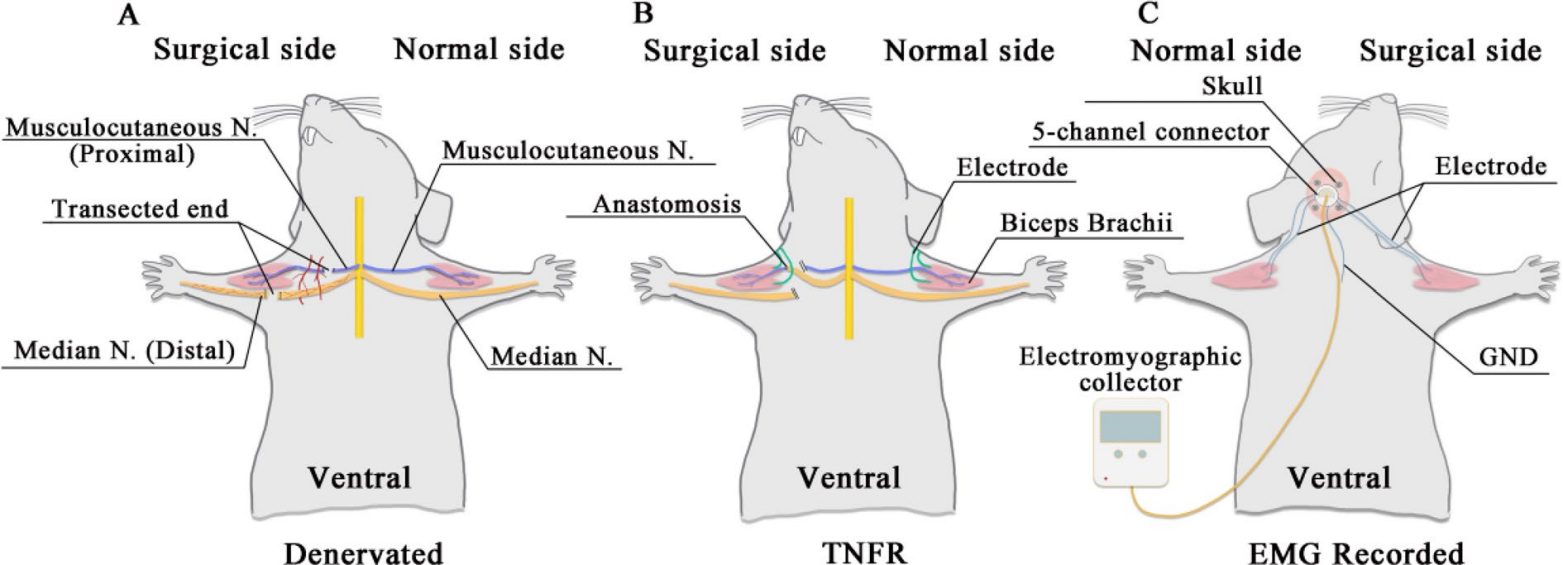
Schematic illustration of the experimental design and EMG recording setup. **A** Denervation procedure illustrating the dissociated proximal end of the musculocutaneous nerve and distal end of the median nerve on the surgical side, with intact neural anatomy on the normal side. **B** TNFR model showing the end-to-end anastomosis between the proximal musculocutaneous nerve and distal median nerve on the surgical side, with bilateral electrode placement in the biceps brachii muscles. **C** EMG recording configuration depicting the 5-channel connector secured to the skull, bilateral muscle electrodes, ground (GND) electrode placement, and connection to the electromyographic collector, shown from the dorsal perspective

A precise 1 mm hole was drilled near the bregma, into which a skull nail was inserted, followed by a five-channel connector pre-welded to Teflon-coated stainless steel electrode wires. This assembly was affixed to the skull using ultraviolet-curing adhesive (Fig. 3). Subsequently, a transverse incision above the biceps brachii muscles facilitated access, allowing for careful dissection and exposure of the muscle bellies down to the elbow joint. Surgical forceps were used to subcutaneously route the electrode wires from the skull implant to each biceps brachii muscle, with approximately 5 mm of Teflon coating stripped from the wire ends before meticulous insertion into the muscle bellies. The electrodes were secured using a No. 8 suture needle, and the procedure was concluded with suturing of the incisions and administration of penicillin for infection prophylaxis (Fig. 4).

### EMG signal acquisition and recording

**Fig. 5:**
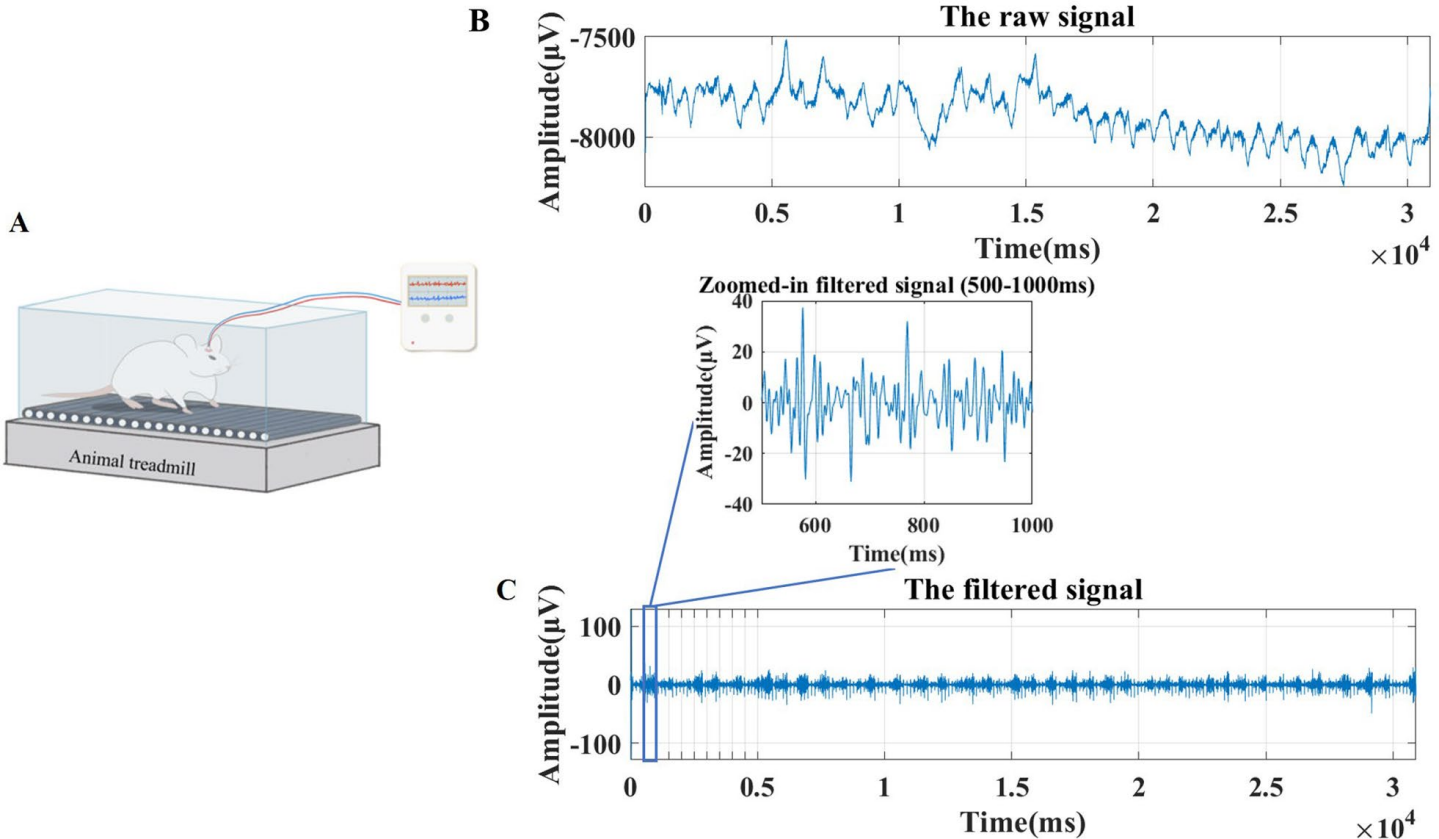
Schematic diagram of an animal treadmill. **A** Schematic diagram of an animal treadmill. The diagram illustrates a rat walking on the treadmill while connected to a self-developed five-channel EMG signal acquisition module via electrode wires implanted in the biceps brachii muscle. The treadmill setup is designed to measure EMG signals during exercise to evaluate muscle function recovery following TNFR surgery. **B** The raw EMG signal recorded from the biceps brachii muscle during treadmill exercise. **C** The filtered EMG signal, showing noise reduction and improved signal clarity after processing. To enhance visualization of waveform details that appear compressed in the full-scale trace, the 500-1000 ms time epoch was extracted and displayed in the inset

EMG signals play a crucial role in evaluating surgical outcomes and assessing functional recovery. Following established methodologies for intramuscular EMG recording and analysis [23, 24], intramuscular EMG signals were recorded using bipolar configuration. Our approach incorporated techniques similar to those described by Boeltz et al. [25] for recording signals from reinnervated muscles, with signal processing methods adapted from studies on EMG analysis in neuromuscular assessment [26–28]. A five-channel connector (Omnetic, Minneapolis, MN, USA) was fixed on the skull with skull nails. Teflon-coated stainless steel wires (Cat# 793500, A-M System Inc, Sequim, WA, USA) were passed subcutaneously to the back (grounded electrode) and both biceps brachii (recording electrodes). After exposing the muscles bilaterally through transverse skin incisions, a 3 mm notch was made in the Teflon coating of the electrodes before implantation. Intramuscular myoelectric signals were collected for 4 weeks starting 1 week after the experimental operation. The electrical stimulation was performed using an invasive approach, with direct surgical exposure of the target median nerve. The EMG signal data were acquired using a self-developed multichannel biopotential signal system at a sampling rate of 1024 Hz (NES-128B01, 128 channels, Shenzhen Institutes of Advanced Technology, Chinese Academy of Sciences, Shenzhen, China). During the EMG recording, rats performed locomotion on a self-developed wheel treadmill (diameter, 0.4 m; circumference, 1.26 m; maximum speed of 30 r/min [0.63 m/s]). For formal experiments, the parameters were set as follows: running time, 30 s; rest time, 30 s; number of cycles, 3 (total time, 3 min); and speed, 9 r/min (0.19 m/s) (Fig. 5).

### EMG signal processing and analysis

The analysis methods were designed to comprehensively assess the intensity, power, and frequency characteristics of EMG signals. First, raw EMG signals were preprocessed by applying a 4th-order Butterworth bandpass filter (20-150 Hz) directly in the time domain to remove motion artifacts and high-frequency noise while preserving physiologically relevant components. This approach was selected over frequency domain filtering as it is more appropriate for real-time bioelectrical signal processing and has become standard practice in EMG analysis. A dual approach combining Short-Time Fourier Transform (STFT) for time-frequency analysis and Root Mean Square (RMS) value calculations was implemented. This approach captures frequency variations over time, providing insights into the dynamic nature of muscle activity post-surgery. The EMG signal amplitude was derived from the STFT results. The STFT was computed as follows:

1 $$S\left(t,f\right)={\int }_{-\infty }^{\infty }x\left(\tau \right)\cdot w\left(\tau -t\right)\cdot {e}^{-j2\pi f\tau }d\tau$$

where $\text{x }(\uptau )$ is the filtered EMG signal (bandpass: 20-150 Hz); $\text{w }(\uptau -\text{t})$ is the window function centered at time t; and the complex exponential.

function. The term x (τ) w (τ − t) represents the windowed signal, which is then.

multiplied by the complex exponential to obtain the frequency representation. The amplitude $A(t,f)$ was then obtained by taking the modulus of the STFT output:

2 $$A(t,f)=|S(t,f)|$$

The parameters for the STFT were chosen thoughtfully to enhance the analysis of the signal characteristics. The window function employed was the Hamming window, chosen for its optimal trade-off between main-lobe width (3.3 dB) and side-lobe suppression (− 42 dB), minimizing spectral leakage compared to rectangular windows. Window length was set to 256 samples (250 ms at 1024 Hz sampling rate). This duration captures 5 cycles of the lowest frequency component (20 Hz) while maintaining quasi-stationarity within each window. Overlap of 50% (128 samples) was implemented to reduce edge effects and improve temporal resolution to 125 ms. Additionally, zero-padding to 512 points enhanced the frequency resolution to 2 Hz. The STFT was computed using MATLAB’s spectrogram function (Signal Processing Toolbox, R2020a) with the aforementioned parameters. The RMS value was utilized to quantify the total energy of the EMG signal, integrating both amplitude and contraction duration. The RMS was calculated using a sliding window approach:

3 $$\text{RMS }=\sqrt{\frac{1}{N}\sum_{i=1}^{N}({x}_{i}^{2})}$$

where N represents the total number of samples in each 1-s window (1024 samples at our sampling rate), and represents the amplitude value of the EMG signal at sample i (unit: μV). The sliding window approach was chosen to provide a continuous measure of the signal’s energy over time, capturing the dynamic changes in muscle activity more effectively than a fixed time window.

For all analyses, we specifically selected the central 20-s period of EMG activity from each 30-s running period to avoid transitional effects at the beginning and end of each running cycle. Within this 20-s stable activity period, RMS values were calculated from consecutive 1-s segments with 50% overlap (0.5-s step size), resulting in 39 overlapping windows for each 20-s period. The frequency band of 40-80 Hz was specifically monitored in the STFT results, as this mid-frequency range has been shown to be most sensitive to changes in motor unit recruitment patterns during recovery [29–31]. This frequency band is particularly sensitive to changes in type II fast-twitch muscle fiber activation, which undergoes significant remodeling following denervation and subsequent reinnervation, making it an ideal neurophysiological marker for tracking functional recovery progression. All signal processing and analyses were performed using MATLAB software (Matlab R2020, MathWorks, Natick, MA, USA). In Fig. 5B and C, the x-axis represents time (milliseconds) and the y-axis represents amplitude (microvolts). The combination of EMG signal amplitude and RMS values provides a balanced assessment of muscle electrical activity, capturing both the instantaneous magnitude and effective intensity of the EMG signals. Specifically, the amplitude derived from the STFT captures the instantaneous intensity of the EMG signals across different frequencies, while the RMS value quantifies the effective intensity of the EMG signals over a given time window. This dual approach ensures a thorough evaluation of muscle function under different experimental conditions.

### Measurement of behavioral function

The post-surgery recovery of phantom limb pain in rats was observed through their autophagy behavior. After collecting intramuscular EMG data, we observed the rats daily for self-injurious behavior. The degree of autophagy in toes is evaluated using a scoring system where a maximum of 13 points can be assigned [32] (Table 1).

**Table 1:** Autophagy scoring system

Score range	Damage level	Presentation	Symbol notation
1–3 points	Level 1	Mild damage: typically seen as nibbling of nails	+
4–6 points	Level 2	Moderate damage: gnawing of two or more distal knuckles	+ +
7–9 points	Level 3	Severe damage: gnawing of three or more distal knuckles or two or more proximal knuckles	+ + +
10–13 points	Level 4	Extremely severe damage: more than three proximal knuckles are gnawed	+ + + +

One point is awarded for damage to one or more toenails; one point is given for damage to the distal segment of each toe, with each affected toe contributing one point; if autophagy occurs in the distal segment of all toes, one point is awarded in total regardless of the number of toes affected; one point is given for damage to the proximal phalangeal segment of each toe, with each affected toe contributing one point; and if autophagy occurs in the proximal phalangeal segment of all toes, one point is awarded in total regardless of the number of toes affected. This system ensures consistent point allocation based on the specific damage or autophagy observed.

### In vivo mechanical analysis of biceps brachii

**Fig. 6:**
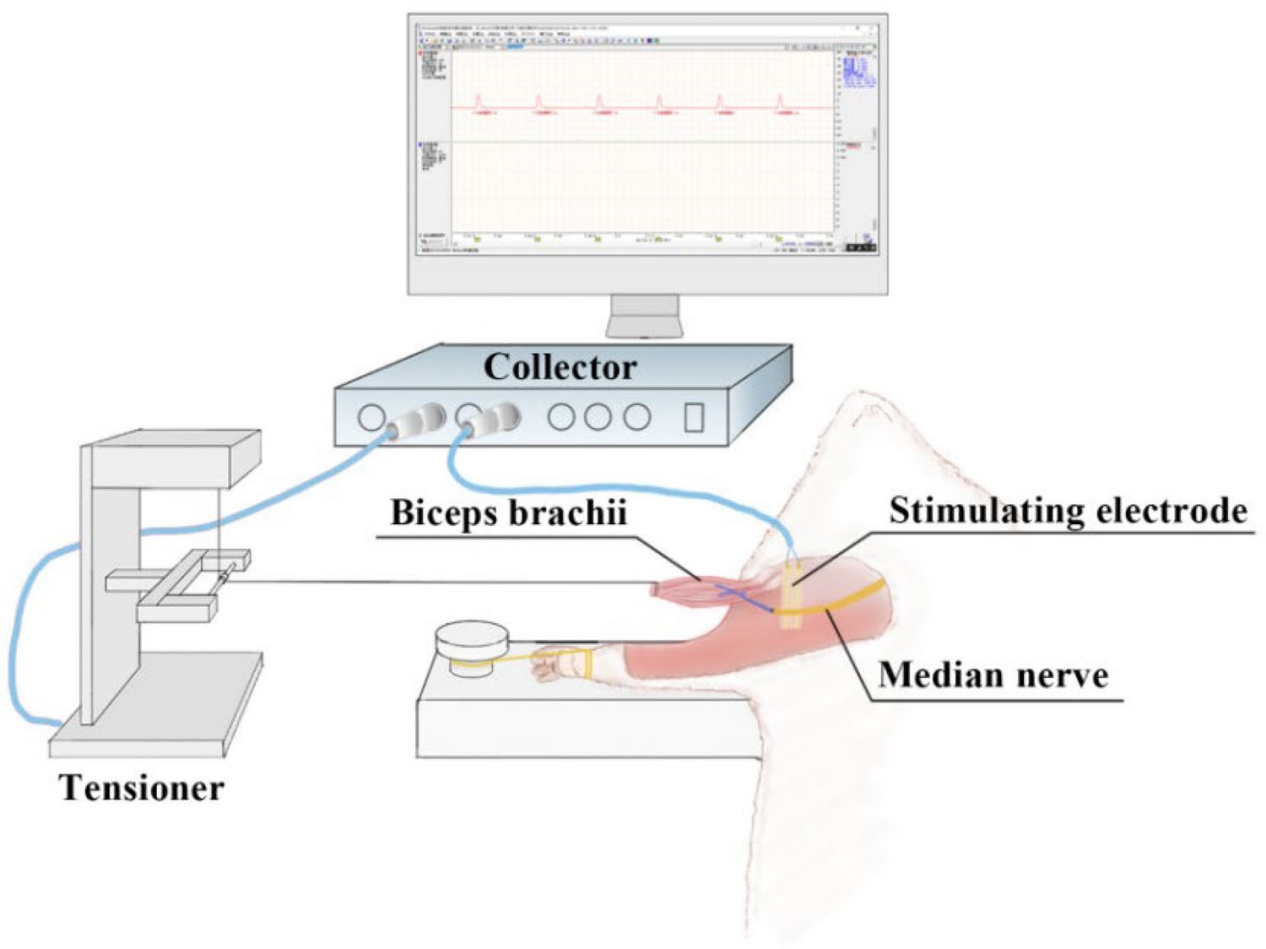
Muscle strength acquisition system

The in vivo mechanical analysis of the biceps brachii muscle provides valuable insights into its functionality under both normal and pathological conditions, and helps in assessing the efficacy of various treatments, including post-surgical recovery [33]. Following the observation of autophagic behaviors, we conducted an examination of the biomechanical properties of the biceps brachii muscle using the Melab-u/8c502 biosignal acquisition system, equipped with a pressure sensor capable of handling up to 50 g (Fig. 6). To ensure accurate measurements, we stimulated the proximal region of the nerve graft. This allowed us to evaluate the functional mimicry between the median nerve and the musculocutaneous nerve subsequent to surgical interventions.

The biceps brachii on the surgical side was stretched and stimulated via the median nerve, with muscle strength recorded under parameters for maximum contraction force (automatic amplitude adjustment, 1-s main cycle, 0.2 ms pulse width, 0 to 10 V amplitude range) and maximum tetanic contraction (automatic frequency adjustment, 1-s main cycle, 0.2 ms pulse width, 5 V initial amplitude, 1 to 50 Hz frequency range).

### Hematoxylin and eosin (H.E.) staining of the DRG

After measuring muscle strength, morphological observations were conducted using Hematoxylin and Eosin (H.E.) staining to examine the dorsal root ganglia (DRG), which are critical structures where sensory neurons converge. This technique allowed us to identify and assess the characteristics of these neurons within the ganglia. Following the in vivo mechanical analysis, the rats were anesthetized with isoflurane and cannulated through the left ventricle to the ascending aorta for perfusion fixation. The spinal cord and DRG of the C5-T1 segment were removed and immediately fixed in 4% paraformaldehyde for 24 h. The samples were dehydrated using a gradient sucrose solution, embedded in an optimal cutting temperature compound, and stored at − 80 ℃. The embedded DRG samples were then sectioned at 20 μm thickness using a Leica CM1950 cryostat, and stained with H.E. stain (ZSGBBio, Beijing, China). The slides were observed under an upright microscope (Eclipse E100, Nikon, Japan) with a 40 × objective lens, and the number of neurons was counted using ImageJ software (National Institutes of Health, Bethesda, MD, USA).

### Immunofluorescence staining of the spinal cord

Finally, the spinal cord was subjected to immunofluorescence staining to assess the distribution and morphology of synapses on motor neurons. Synaptophysin (SYN) and postsynaptic density protein 95 (PSD-95) are pivotal proteins that modulate neuromuscular junction function and play crucial roles in nerve signal transmission and muscle action [34]. Spinal cord tissues embedded in paraffin were sectioned at 15-20 μm thickness using a Leica cryostat at − 20 ℃. These sections were then deparaffinized, rehydrated, and subjected to antigen retrieval.

The sections were blocked with bovine serum albumin and incubated with primary antibodies against SYN (rabbit anti-SYN antibody, 1:500, Cat# bs-23504R, RRID: AB_2895150, Bioss) and PSD-95 (mouse-produced primary antibody, specific catalog and concentration to be added). Following primary antibody incubation, the sections were incubated with fluorescently labeled secondary antibodies: goat anti-rabbit IgG/Cy3 for SYN (green fluorescence) and goat anti-mouse IgG conjugated to Alexa Fluor 594 for PSD-95 (red fluorescence). After nuclear counterstaining with 4’, 6-diamidino-2-phenylindole (DAPI, Abcam), the sections were mounted and observed under an upright fluorescence microscope (E100, Nikon) using a 40 × objective lens. The mean optical density of SYN and PSD-95 was quantified using ImageJ software (National Institutes of Health).

### Statistical analysis

Functional and histopathological analyses were performed under blinding protocols to ensure unbiased results. The normality test was performed using the Shapiro–Wilk test with a significance level of 0.05. Homogeneity of variances was confirmed with Levene’s test. All data samples conformed to normal distributions (P < 0.05). We present quantitative data as means ± standard deviation. Differences between experimental groups were assessed using one-way ANOVA, followed by post-hoc Bonferroni tests for all multiple comparisons. This approach was selected to control for Type I error rates across all analyses. A p-value of less than 0.05 was considered statistically significant. All statistical analyses were conducted under blinding protocols using GraphPad Prism software version 9.0.0 (GraphPad Software, San Diego, CA, USA).

## Results

### General conditions of rats

Post-surgery, all rats exhibited normal feeding behavior and showed no signs of infections, ulcers, or nervous system disorders.

### Effect of TNFR surgery on intramuscular EMG signal analysis in rats

**Fig. 7:**
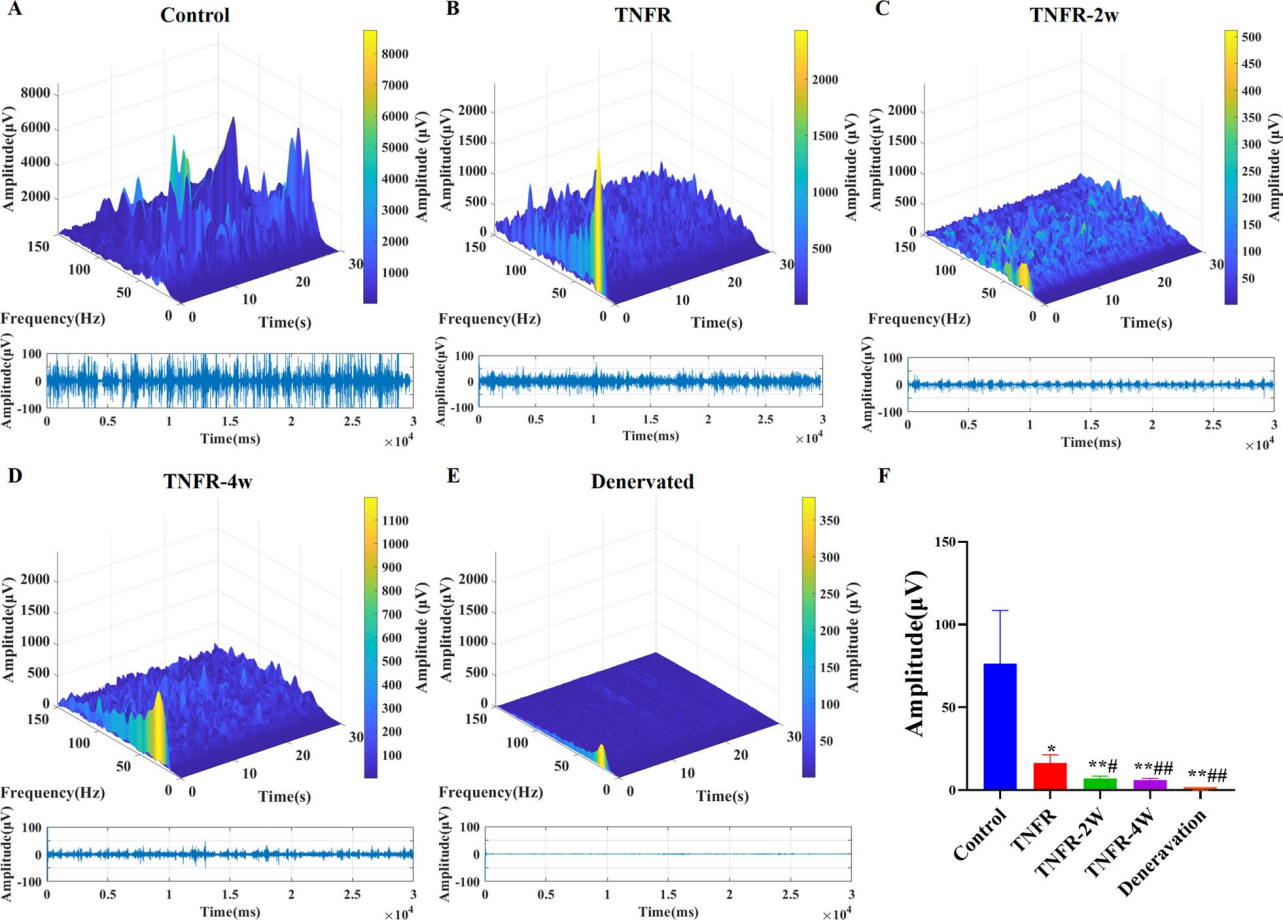
Intramuscular EMG signal amplitude analysis across experimental groups. Representative EMG signal recordings for each experimental group 4 weeks after intervention are shown in 3D spectrograms (top) and 2D time-amplitude plots (bottom): **A** The control group with normal innervation showed robust, high-amplitude EMG activity. **B** the immediate TNFR group showed a significant recovery of the EMG signal pattern. **C** the TNFR-2W group showed moderate recovery with a 2-week delay in intervention. **D** the TNFR-4W group showed similar moderate recovery despite a 4-week delay in intervention. **E** the denervated group showed only background electrical noise with no functional EMG activity. **F** Quantitative comparison of EMG signal amplitudes among groups. Data are presented as mean ± SD (n = 6 per group). Statistical analysis: One-way ANOVA (F (4, 70) = 16.40, P < 0.0001) followed by Bonferroni multiple comparison test. *P < 0.05, **P < 0.01 compared with the control group.# P < 0.01,##P < 0.0001 compared with the TNFR group. *TNFR* targeted nerve function replacement, *TNFR-2W* 2-week delayed TNFR group, *TNFR-4W* 4-week delayed TNFR group, and *SD* standard deviation.

**Fig. 8:**
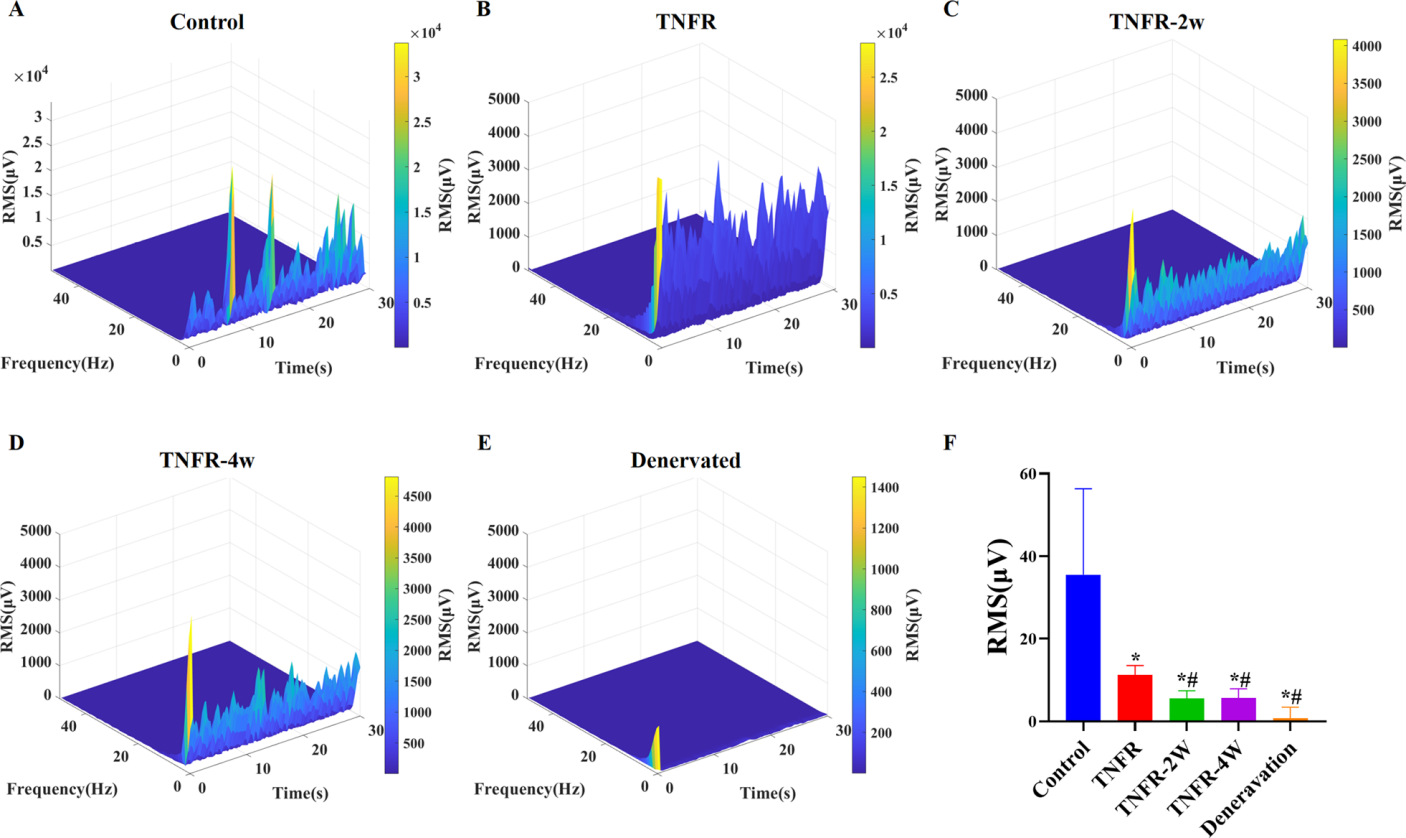
EMG signal RMS analysis across experimental groups. **A**-**E** 3D time-frequency diagrams of RMS values from each experimental group at 4 weeks post-intervention: **A** Control group showing robust signal presence with high RMS value across frequency spectrum. **B** TNFR group demonstrating reduced but substantial RMS values with moderately preserved signal presence across frequencies. **C** and **D** TNFR-2W and TNFR-4W groups showing significantly reduced RMS values with limited signal across frequencies. **E** Denervated group displaying only baseline noise with negligible RMS values. **F** Quantitative comparison of EMG signal RMS values across groups. Data are expressed as mean ± SD (n = 6 per group). Statistical analysis: one-way ANOVA (F (4, 70) = 8.886, P < 0.0001) followed by Bonferroni multiple comparisons test. *P < 0.0001 compared to control group.# P < 0.0001 compared to TNFR group. *TNFR* targeted nerve function replacement, *TNFR-2W* 2-week delayed TNFR group, *TNFR-4W* 4-week delayed TNFR group, and *SD* standard deviation

EMG signals were successfully collected from rats 4 weeks post-surgery. Figures 7A-E show the EMG signal amplitude analysis and Figs. 8A-E show the EMG signal RMS analysis from all groups (control, TNFR, TNFR-2W, TNFR-4W and denervated), respectively. The EMG signals from the TNFR, TNFR-2W, and TNFR-4W groups were analyzed following TNFR surgery (Figs. 7B-D and 8B-D). Both the EMG signal amplitude and RMS values in the immediate TNFR group were significantly higher than those in the other two experimental groups (TNFR-2W and TNFR-4W). In stark contrast, the denervated group exhibited only background noise with no detectable EMG signals (Figs. 7E and 8E), confirming complete loss of neuromuscular activity. The comprehensive statistical analysis of intramuscular signal parameters across all experimental conditions is presented in Figs. 7F and 8F, which display the quantitative amplitude and RMS values, respectively. These analyses clearly demonstrate the superior functional recovery achieved with immediate TNFR intervention compared to delayed procedures. Significant differences were observed in the EMG signal amplitude and RMS values among the groups (Figs. 7F and 8F). The EMG signal amplitude and RMS values were significantly higher in the control group compared to the TNFR group (P < 0.05). Within the TNFR groups, the EMG signal amplitude and RMS values were higher in the immediate TNFR group than in the TNFR-2W and TNFR-4W groups (P < 0.05). EMG signal amplitude and RMS values were significantly lower in the denervated group compared to the other groups (P < 0.05). While there was no significant difference in EMG signal amplitude and RMS values between the TNFR-2W and TNFR-4W groups (P > 0.05), both groups exhibited EMG signal amplitude and RMS values significantly higher than those of the denervated group (P < 0.05).

### Autotomy behavior observation

**Fig. 9:**
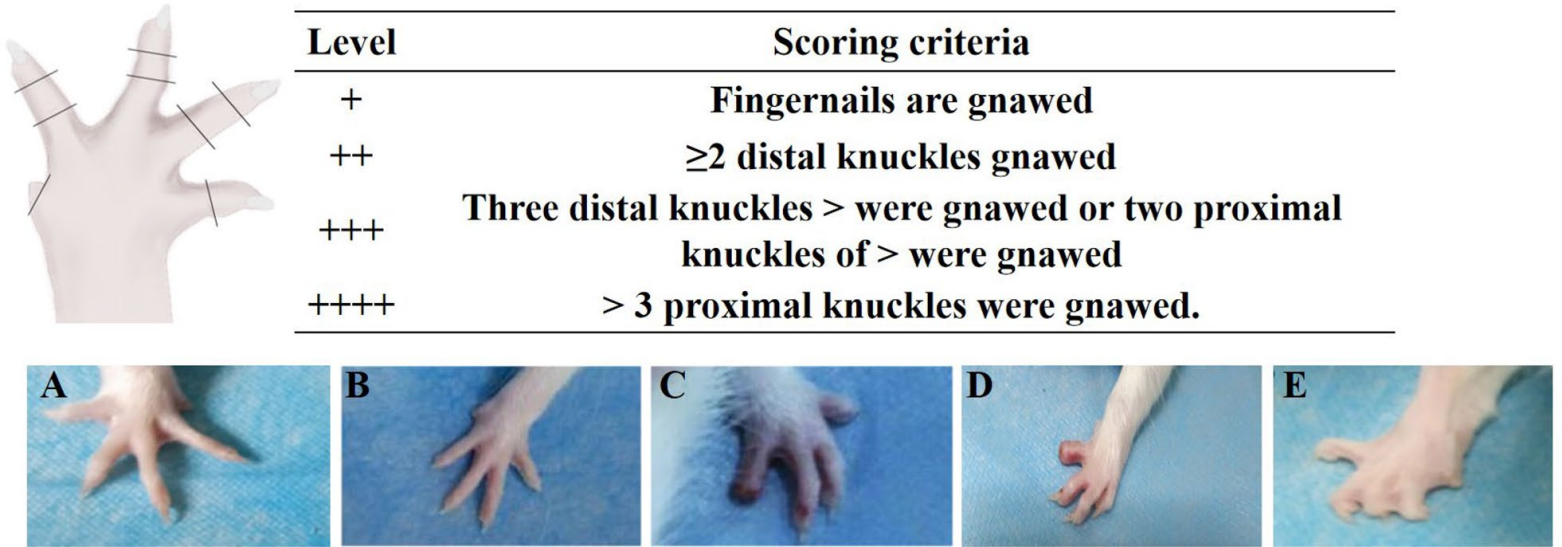
Conditions of the autophagic upper limbs in rats. **A** Control group showed no autophagy. **B** TNFR group showed no autophagy. **C** TNFR-2W group showed level 1 (mild) autophagy. **D** TNFR-4W groups showed level 1 (mild) autophagy. **E** denervated group showed level 4 (extremely severe) autophagy. *TNFR* targeted nerve function replacement, *TNFR-2W* 2-week delayed TNFR group, and *TNFR-4W* 4-week delayed TNFR group

The experimental results indicated that the control and TNFR groups displayed no abnormalities in toe morphology. However, in the denervated group, phantom limb pain and abnormal autophagy behavior were observed as early as one week post-surgery, with symptoms intensifying over time (Fig. 9). The TNFR-2W and TNFR-4W groups exhibited autophagy, redness, and swelling of the toes at one week post-surgery, but these symptoms, along with the phantom limb pain, decreased and eventually subsided within 14-21 days. Table 2 shows the autotomy behavior in rats across different time points for each experimental group. In the TNFR-2W group, 3 rats exhibited level 1 autotomy behavior in Week 1 while 3 had no symptoms; 4 rats showed level 2 behavior in Week 2 with 2 remaining asymptomatic; by Week 3, only 2 rats still displayed level 1 behavior with 4 recovered to no symptoms; and by Week 4, all 6 rats had completely recovered with no symptoms. The TNFR-4W group showed a similar recovery pattern, with 3 rats displaying level 1 behavior in Week 1, progressing to 4 rats with level 2 behavior in Week 2, then improving to 4 rats with level 1 behavior in Week 3, and finally all 6 rats showing complete recovery by Week 4. In contrast, rats in the denervated group demonstrated progressively worsening symptoms, with 5 rats showing level 1 autotomy in Week 1, all 6 rats reaching level 2 in Week 2, and all 6 rats progressing to severe level 3 autotomy behavior by Weeks 3 and 4. This demonstrates that without intervention, denervated rats experienced continual deterioration in symptoms throughout the observation period. Additionally, by the end of the observation period, the toe morphology in the TNFR intervention groups (TNFR-2W and TNFR-4W) appeared similar to that observed in the control group, suggesting resolution of the initial autotomy symptoms.

**Table 2:** Autophagy of rats in each group

Groups	Symptom Level	Week1	Week 2	Week 3	Week 4
Control group	No symptoms	6	6	6	6
	Level 1 ( +)	0	0	0	0
	Level 2 (+ +)	0	0	0	0
	Level 3 (+ + +)	0	0	0	0
TNFR group	No symptoms	6	6	6	6
	Level 1 ( +)	0	0	0	0
	Level 2 (+ +)	0	0	0	0
	Level 3 (+ + +)	0	0	0	0
TNFR-2W	No symptoms	3	2	4	6
	Level 1 ( +)	3	0	2	0
	Level 2 (+ +)	0	4	0	0
	Level 3 (+ + +)	0	0	0	0
TNFR-4W	No symptoms	3	2	2	6
	Level 1 ( +)	3	0	4	0
	Level 2 (+ +)	0	4	0	0
	Level 3 (+ + +)	0	0	0	0
Denervated	No symptoms	1	0	0	0
	Level 1 ( +)	5	0	0	0
	Level 2 (+ +)	0	6	0	0
	Level 3 (+ + +)	0	0	6	6

The numbers in parentheses represent the number of animals exhibiting autophagy behavior in each group per week

*TNFR* targeted nerve function replacement, *TNFR-2W* 2-week delayed TNFR group, *TNFR-4W* 4-week delayed TNFR group

### Effect of TNFR surgery on biceps brachii contraction force

**Fig. 10:**
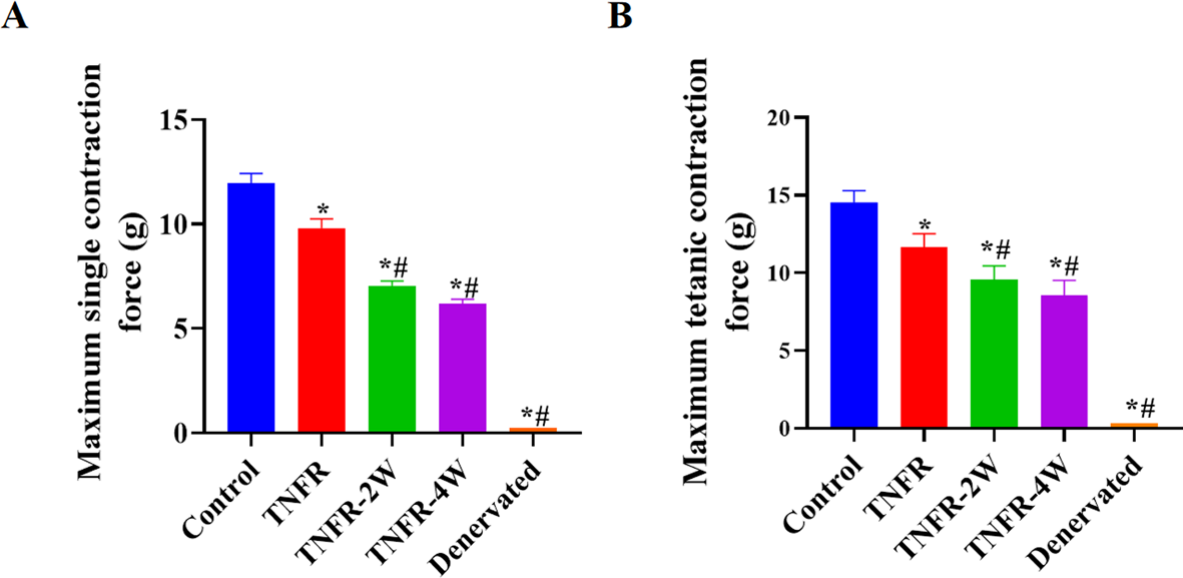
Biceps contraction force of each group. Maximum single contraction force (**A**) and maximum tetanic contraction force (**B**) across all experimental groups. Data are expressed as mean ± SD, n = 6 per group. Statistical analysis: one-way ANOVA (F (4, 25) = 31.24, P < 0.01) followed by Bonferroni multiple comparisons test. *indicates the difference between the control group and other groups, *P < 0.05; # indicates the difference between TNFR and other groups, # P < 0.01. *TNFR* targeted nerve function replacement, *TNFR-2W* 2-week delayed TNFR group, *TNFR-4W* 4-week delayed TNFR group, and *SD* standard deviation

Four weeks post-surgery, both the maximum contraction force and the maximum tetanic contraction force of the biceps brachii were significantly higher in the control group compared to the denervated, TNFR, TNFR-2W, and TNFR-4W groups (P < 0.05) (Fig. 10). Furthermore, the immediate TNFR group showed significantly greater muscle strength than the TNFR-2W, TNFR-4W, and denervated groups (P < 0.05). However, there was no statistically significant difference in muscle strength between the TNFR-2W and TNFR-4W groups (P > 0.05), though both were significantly stronger than the denervated group (P < 0.05).

### Effects of TNFR surgery on the DRG neurons in rats

**Fig. 11:**
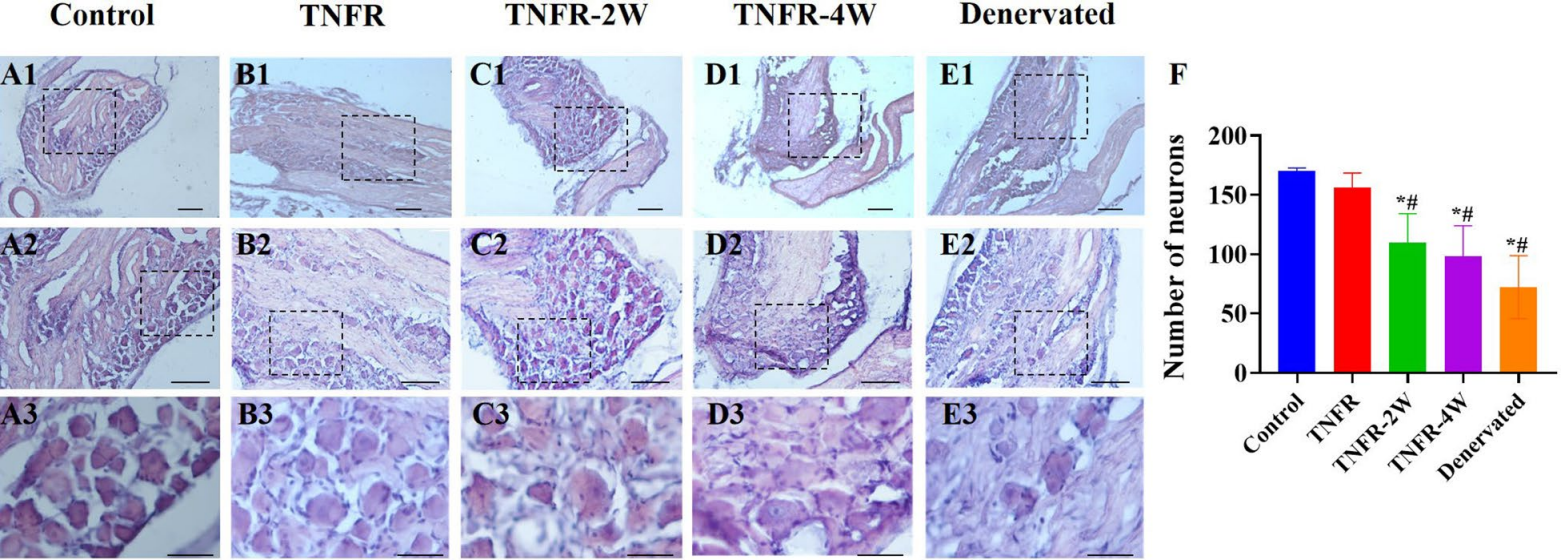
H.E. staining of DRG of C5-T1 segment of the right forelimb in all the groups. **A1**-**E1** DRG sections at 4 × magnification (scale bar: 500 μm): Control, TNFR, TNFR-2W, TNFR-4W, and Denervated groups. **A2**-**E2** Magnified images (10 × objective, scale bar: 200 μm) of the same groups. **A3**-**E3** Further magnified images (40 × objective lens, scale bar: 50 μm) showing detailed neuronal morphology. **F** Quantitative analysis of the number of sensory neurons across groups. Data are expressed as mean ± SD, n = 6 per group. Statistical analysis: one-way ANOVA (F (4, 25) = 13.64, P < 0.05) followed by Bonferroni multiple comparisons test. *indicates the difference between the control group and other groups, *P < 0.05; # indicates the difference between TNFR and other groups, # P < 0.05. *TNFR* targeted nerve function replacement, *TNFR-2W* 2-week delayed TNFR group, *TNFR-4W* 4-week delayed TNFR group, and *SD* standard deviation

H.E. staining was performed on the DRG samples of each group. The number of sensory neurons was significantly higher in the TNFR group than in the TNFR-2W and TNFR-4W groups (P < 0.05; Fig. 11). The TNFR-2W group showed a trend toward better preservation of neuronal morphology compared to the TNFR-4W and denervated groups, although this difference was not statistically significant (P > 0.05).

### Effects of TNFR surgery on SYN and PSD-95 expression in the spinal cord motor neurons in rats

**Fig. 12:**
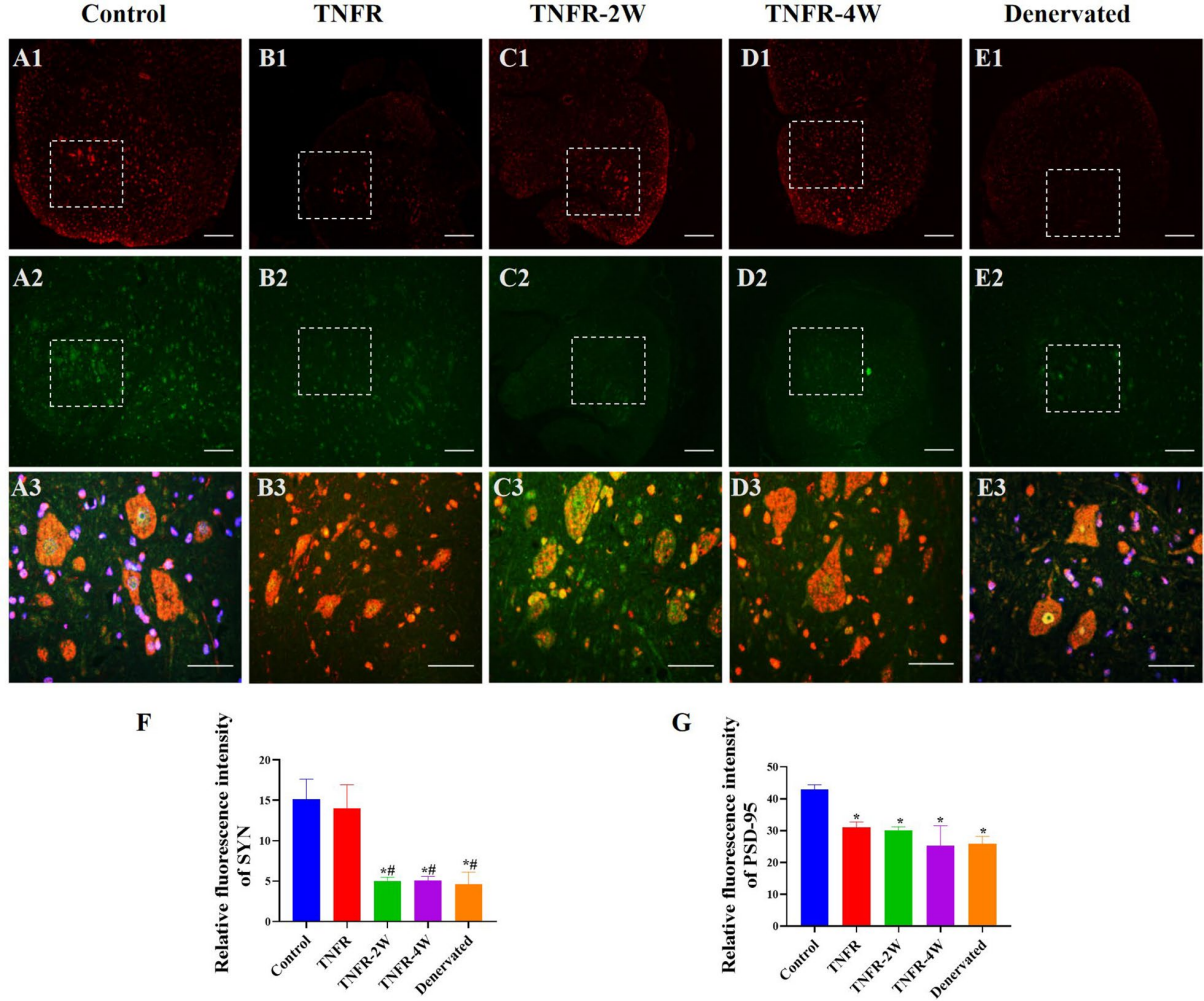
SYN and PSD-95 expression in the motor neurons in the anterior horn of the spinal cord in rats. **A1**-**E1** Representative immunofluorescence staining images of SYN (red) in the Control, TNFR, TNFR-2W, TNFR-4W, and Denervated groups (4 × objective lens, scale bar: 200 μm). **A2**-**E2** PSD-95 staining (green) in the same groups (4 × objective lens, scale bar: 200 μm). **A3**-**E3** Merged images showing co-localization of SYN and PSD-95 in the boxed areas (40 × objective lens, scale bar: 50 μm). Quantitative analysis of **F** relative fluorescence intensity of SYN and **G** relative fluorescence intensity of PSD-95 across groups. Data are expressed as mean ± SD, n = 6 per group. Statistical analysis: one-way ANOVA (SYN: F (4, 25) = 18.72, P < 0.0001; PSD-95: F (4, 25) = 31.24, P < 0.0001) followed by Bonferroni multiple comparisons test. *indicates the difference between the control group and other groups, *P < 0.05; # indicates the difference between TNFR and other groups, # P < 0.05. *SYN* synaptophysin, *PSD-95* post-synaptic density protein 95, *TNFR* targeted nerve function replacement, *TNFR-2W* 2-week delayed TNFR group, *TNFR-4W* 4-week delayed TNFR group, and *SD* standard deviation

The immunofluorescence intensity of the motor neurons in the anterior horn of the spinal cord was significantly stronger in the control group compared to all experimental groups. Compared with the control group, the SYN immunoreactivity in the TNFR group showed a reduction (P > 0.05; Fig. 12F). The SYN immunoreactivity was significantly higher in the TNFR group compared to the TNFR-2W, TNFR-4W, and denervated groups (P < 0.05; Fig. 12F), demonstrating better preservation of presynaptic terminals with immediate intervention. For PSD-95 expression, all experimental groups (TNFR, TNFR-2W, TNFR-4W, and denervated groups) showed significantly reduced levels compared to the control group (P < 0.05; Fig. 12G). Importantly, there were no significant differences in PSD-95 immunoreactivity among all experimental groups (P > 0.05; Fig. 12G), indicating that intervention timing had no differential impact on postsynaptic protein expression. While fluorescence intensity provides valuable quantitative and spatial information on protein expression, it may be influenced by external factors such as staining time and antibody concentration. To mitigate these potential variables, we implemented rigorous methodological controls including standardized staining protocols, consistent antibody lots, uniform incubation times, and calibrated image acquisition settings across all experimental groups. This standardization ensures that the observed differences in SYN and PSD-95 expression patterns (Fig. 12) accurately reflect biological changes rather than technical variations.

## Discussion

Our experiment demonstrates that immediate nerve reinnervation following injury significantly improves muscle function, showing markedly better results compared to reinnervation delayed by 2 and 4 weeks. Additionally, we found that combining implanted electrodes with TNFR is an effective method for in vivo tracking of functional recovery. Our research confirms the feasibility of functional restoration through TNFR surgery, supported by both bio-structural and EMG data as shown in Figs. 7 and 8, which is crucial for the development of myoelectric prosthetics. Early detection of signals from the target muscle post-surgery indicates a significant reduction in overall functional recovery time. These findings provide important evidence for further optimizing nerve reinnervation surgery.

TMR can significantly enhance axon regeneration, increase the quantity and size of regenerated axons, shorten the duration of muscle reinnervation, and prevent neuromas [35–37]. In contrast, our TNFR approach offers distinct advantages by maintaining original neural pathways while providing supplementary neural input. In this work, we strategically placed the site for neurorrhaphy close to the target muscle to reduce both the regeneration distance and duration as illustrated in Fig. 4. To achieve superior results, we made several refinements to the experimental protocol. Initially, we used a pull hook to elevate the pectoralis major, pectoralis minor, and portions of the deltoid. Although this could have prevented muscle trauma, it did not provide a satisfactory view of the operative site. Therefore, we opted to carefully separate the pectoralis major and minor muscles to obtain a clear view of the operation site, reducing surgical time despite its unfavorable effect on motor function. Regarding the surgical approach, following guidelines from a previous study [38], we ensured optimal conditions for nerve reinnervation. By incising the musculocutaneous nerve (MCN) proximally and the median nerve (MN) distally, we increased nerve length, facilitating a tension-free neurorrhaphy as shown in Fig. 2C. We refined our surgical procedure to prevent damage to the local vascular network and limit nerve disconnection to only the necessary segments for ligation. This approach effectively reduced bleeding and enhanced blood supply to the transected nerve, crucial for delivering nutrients and regeneration factors [39]. Neovascularization served as a conduit for the restored nerve, directing regenerating axons [40–42] and significantly reducing recovery time. We performed second-stage operations with remarkable precision and ease. The surgical field was unobstructed, allowing for direct and effortless nerve access. This streamlined approach resulted from meticulous planning and execution, ensuring minimal tissue manipulation to effectively expose target areas. Consequently, this may have contributed to reduced fibroblast infiltration and lower damage to blood vessels.

For functional assessment, the treadmill examination is a streamlined and precise method to regulate and assess the behavioral parameters of rats during training regimens [42, 43]. In this project, we used a customized wheel treadmill as depicted in Fig. 5A, unlike conventional flat treadmills, to enhance engagement and activity in the forelimbs. Our preliminary tests revealed that the wheel treadmill prevented the rats from leaping forward using their hind limbs, thereby compelling increased reliance on and participation of the forelimbs. After a brief training period, the rats demonstrated improved balance and active participation on the wheel treadmill. This approach provided an effective strategy for observing and assessing the recovery trajectory of forelimb muscle functionality and neural control.

To validate this method, we also measured the maximum contraction force and maximum tetanic contraction force of the biceps brachii muscle presented in Fig. 10A, B, which helped assess the function and health status of the muscle fibers. In the immediate TNFR group, the application of TNFR following an injury resulted in the highest EMG signal amplitude, RMS values, maximum contraction force, and maximum tetanic contraction force compared with the other experimental groups. In contrast, both EMG signal amplitude and RMS values significantly decreased in the delayed TNFR groups, specifically the TNFR-2W and TNFR-4W groups. Therefore, immediate TNFR preserved significantly better muscle function compared with delayed intervention.

Irreversible muscle atrophy post-denervation, as described by Soendenbroe [44], can lead to severe muscle weakness and unfavorable functional prognoses. This accounts for the poor performance observed in the denervated group during functional assessments, where all three evaluation indices were lower than those of the other groups as demonstrated in Figs. 7F, 8F, and 10A, B. The delayed TNFR groups showed no significant differences in their EMG signal amplitude, RMS values, and muscle contraction force, consistent with the results of the functional assessment. These results indicate the significant benefits of TNFR, especially immediate TNFR intervention, for effective restoration of motor function.

Our findings on the timing-dependent efficacy of nerve reinnervation parallel recent evidence in related fields. A review by Dominguez et al. [45] demonstrated similar timing-dependent effects with TMR, where acute TMR showed superior pain management outcomes compared to delayed procedures, further supporting the critical importance of early intervention in neural pathway restoration.

Regarding behavioral outcomes, while phantom limb pain typically occurs in individuals following limb amputation, the observed self-mutilation behaviors (or autotomy) in our study may indicate neuropathic pain or sensory disturbances associated with delayed nerve repair rather than phantom pain. Autotomy behaviors were observed in rats that underwent delayed surgeries, which involved nerve severance, while those receiving immediate surgery did not exhibit these signs as documented in Fig. 9 and Table 2. Autotomy behaviors rarely follow median nerve injury [46, 47]. We selected Sprague Dawley rats for their availability and established use in nerve injury models. However, Carr et al. [48], noted this strain shows higher rates of autotomy after injury compared to Lewis rats, which exhibit much lower rates of this behavior. Future studies might consider using alternative strains with lower susceptibility to autotomy to further minimize these effects and clarify if the autophagy behavior observed is strain-specific or model-dependent. These results suggest that TNFR intervention yields the best results in nerve reconstruction.

For histological findings, DRG are composed of sensory fiber cells that receive all nerve impulses, including general somatosensory and visceral sensations, from the body’s receptors. These impulses are then relayed to the spinal cord via sensory fibers. In this study, we used H.E. staining to examine the morphology and number of sensory neurons in the DRG as shown in Fig. 11A1-E1. Our analysis revealed that TNFR intervention effectively normalized neuronal morphology, restoring the size of the cell bodies and the number of axons to levels statistically similar to those in the control group. However, delayed TNFR treatment in the TNFR-2W and TNFR-4W groups only partially recovered sensory neuron morphology and functionality, with observed instances of demyelination. Quantitative analysis (Fig. 11F) confirmed a significant reduction in sensory neuron numbers in the TNFR-2W and TNFR-4W groups compared to TNFR. Therefore, timely TNFR intervention, particularly when implemented immediately, is essential for the effective recovery of sensory neurons in the DRG.

Regarding synaptic marker analysis, SYN protein is localized to synaptic vesicles in the presynaptic terminals of neurons. It is involved in the release of activity-dependent neurotransmitters and plays a crucial role in synaptic plasticity. In contrast, PSD-95 is essential for post-synaptic signal transduction and synaptic plasticity by anchoring receptor proteins at the synaptic membrane [49, 50] Alterations in PSD-95 expression in motor neurons can affect synaptic strength, contributing to changes in motor activity. Our results showed a significant decrease in the expression of SYN and PSD-95 across all experimental groups compared to the control group as visualized in Fig. 12A1-E3. Specifically, SYN, a presynaptic vesicle protein essential for neurotransmitter release, showed relatively better restoration in the TNFR group compared to the TNFR-2W, TNFR-4W, and denervated groups.

However, PSD-95, a scaffolding protein critical for postsynaptic organization and signaling, remained similarly reduced across all intervention groups as quantified in Fig. 12F-G, suggesting differential effects on pre- and postsynaptic markers.

This pattern indicates that while TNFR intervention may partially preserve presynaptic structures (as evidenced by improved SYN expression), postsynaptic architecture (represented by PSD-95) appears more resistant to recovery regardless of intervention timing. The persistent reduction in PSD-95 across all experimental groups suggests that postsynaptic densities in motor neurons within the anterior horn of the spinal cord may require additional interventions beyond TNFR to achieve complete recovery. This differential response between presynaptic and postsynaptic markers highlights the complex nature of synaptic reorganization following peripheral nerve injury and suggests that comprehensive neuronal recovery may require targeted approaches that address both pre- and postsynaptic elements of the neural circuit.

## Limitations

This study has several important limitations. Our use of a rat model presents inherent translational challenges to human applications due to differences in nerve regeneration capacity, immune responses, and functional recovery patterns between species. The sample size (n = 6 per group), while statistically adequate based on our power analysis, may limit detection of subtle differences between intervention timepoints. The follow-up period of 4 weeks, while sufficient to demonstrate significant differences between immediate and delayed interventions, may not capture the full trajectory of recovery that could occur over longer timeframes. Additionally, our assessment methods, while comprehensive, could not evaluate all aspects of sensorimotor function. The EMG electrode implantation itself may have influenced local tissue responses and potentially affected outcomes. The autotomy behavior observed predominantly in the denervated and delayed intervention groups represents a complex response that may conflate pain, sensory abnormalities, and other factors, making direct interpretation challenging. Furthermore, while we demonstrated functional and histological improvements with TNFR, the underlying molecular mechanisms remain largely unexplored. Finally, our single-nerve model (median to musculocutaneous nerve) represents a simplified scenario compared to the complex multi-nerve injuries often seen in clinical settings. Future studies should address these limitations through longer follow-up periods, exploration of molecular mechanisms, and testing in models that more closely approximate clinical scenarios.

## Conclusion

This study demonstrated that TNFR effectively promotes functional recovery following nerve injury in rats, with immediate intervention yielding significantly better outcomes than delayed procedures. Our comprehensive evaluation revealed superior EMG signals, reduced autophagic behavior, and better preservation of muscle function and neuronal structures with TNFR.

Our findings have direct clinical implications for surgical decision-making in trauma and peripheral nerve injury cases. The significant superiority of TNFR over interventions delayed by even 2 weeks suggests that early surgical planning should be prioritized whenever possible. This is particularly relevant for complex trauma cases where multiple surgical procedures may be required. For unavoidable delays, our results showing partial recovery even with 4-week delays provide evidence that TNFR remains beneficial despite suboptimal timing, though expectations for functional outcomes should be appropriately adjusted.

## Supplementary Information

Supplementary file 1.

## Data Availability

The datasets generated and analyzed during the current study are available from the corresponding author upon reasonable request.

## Abbreviations

ANOVA: Analysis of variance
ARRIVE: Animal research: reporting of in vivo experiments
DRG: Dorsal Root Ganglion
EMG: Electromyography
GND: Ground
H.E.: Hematoxylin and eosin
MCN: Musculocutaneous nerve
MN: Median nerve
PSD-95: Postsynaptic density protein 95
RPNI: Regenerative peripheral nerve interfaces
RMS: Root mean square
SPF: Specific pathogen-free
STFT: Short-time fourier transform
SYN: Synaptophysin
TMR: Targeted muscle reinnervation
TNFR: Targeted nerve function replacement
TNFR-2W: 2-Week delayed targeted nerve function replacement
TNFR-4W: 4-Week delayed targeted nerve function replacement
